# Role of Chemerin in Cardiovascular Diseases

**DOI:** 10.3390/biomedicines10112970

**Published:** 2022-11-18

**Authors:** Mirjana T. Macvanin, Manfredi Rizzo, Jelena Radovanovic, Alper Sonmez, Francesco Paneni, Esma R. Isenovic

**Affiliations:** 1 Department of Radiobiology and Molecular Genetics, VINČA Institute of Nuclear Sciences—National Institute of the Republic of Serbia, University of Belgrade, 11000 Belgrade, Serbia; 2 Department of Internal Medicine and Medical Specialties (DIMIS), Università degli Studi di Palermo (UNIPA), 90128 Palermo, Italy; 3 Department of Endocrinology and Metabolism, Gulhane School of Medicine, University of Health Sciences, Ankara 34668, Turkey; 4 University Heart Center, University Hospital Zurich, 8091 Zurich, Switzerland; 5 Center for Translational and Experimental Cardiology (CTEC), Department of Cardiology, University Hospital Zurich, University of Zurich, Wagistrasse 12, 8952 Schlieren, Switzerland

**Keywords:** chemerin, cardiovascular disease, chemerin receptors, adipokine, inflammation, endothelial dysfunction, chemerin-targeting therapeutic agents

## Abstract

(1) Background: Obesity is closely connected to the pathophysiology of cardiovascular diseases (CVDs). Excess fat accumulation is associated with metabolic malfunctions that disrupt cardiovascular homeostasis by activating inflammatory processes that recruit immune cells to the site of injury and reduce nitric oxide levels, resulting in increased blood pressure, endothelial cell migration, proliferation, and apoptosis. Adipose tissue produces adipokines, such as chemerin, that may alter immune responses, lipid metabolism, vascular homeostasis, and angiogenesis. (2) Methods: We performed PubMed and MEDLINE searches for articles with English abstracts published between 1997 (when the first report on chemerin identification was published) and 2022. The search retrieved original peer-reviewed articles analyzed in the context of the role of chemerin in CVDs, explicitly focusing on the most recent findings published in the past five years. (3) Results: This review summarizes up-to-date findings related to mechanisms of chemerin action, its role in the development and progression of CVDs, and novel strategies for developing chemerin-targeting therapeutic agents for treating CVDs. (4) Conclusions: Extensive evidence points to chemerin’s role in vascular inflammation, angiogenesis, and blood pressure modulation, which opens up exciting perspectives for developing chemerin-targeting therapeutic agents for the treatment of CVDs.

## 1. Introduction

Cardiovascular disease (CVDs) represents the major global cause of death and disability in humans, accounting for approximately one-third of all deaths worldwide [1]. The close relationship between CVDs and obesity is well documented. It points to the connection between the excessive accumulation of visceral fat and the clustering of metabolic diseases, such as dyslipidemia, type 2 diabetes, and hypertension, which eventually culminate in the development of CVDs [2]. Obesity is associated with many metabolic abnormalities that disrupt cardiovascular homeostasis by stimulating inflammatory processes that recruit immune cells to the injury site and reduce nitric oxide (NO) levels, resulting in increased blood pressure, endothelial cell migration, proliferation, and apoptosis [3]. In addition to its role in storing excess fat, adipose tissue also acts as an endocrine organ that produces numerous biologically active, cytokine-like peptides called adipokines that can elicit autocrine, paracrine, and endocrine functions in the body [4]. Adipokines regulate adipose tissue metabolism, differentiation, and energy balance storage and are essential for normal physiological functioning [5]. In addition, adipokines may alter immune responses, lipid metabolism, insulin sensitivity, vascular homeostasis, and angiogenesis, thus directly or indirectly affecting CVDs pathogenesis [6,7]. It has been acknowledged that dysfunctional adipose tissue remodelling results in adipokine disbalance, leading to systemic inflammation that affects the cardiovascular endothelium, resulting in hypertension and endothelial cell proliferation [6,8,9]. Numerous other adipokines whose expression has been shown to be upregulated in the obese state have been identified since the discovery of leptin, an adipose-specific adipokine with a central role in regulating food intake and energy expenditure. Tumour necrosis factor (TNF)-α, interleukin (IL)-6, IL-1β, and resistin belong to a pro-inflammatory group of adipokines that lead to exacerbation of metabolic and cardiovascular diseases [10]. However, some adipokines that are downregulated in obesity have anti-inflammatory properties, thus exerting a protective function against conditions associated with obesity, including CVDs [11].

In this review, we focus on the role of chemerin in the pathophysiology of CVDs. Chemerin is an adipokine with multiple roles in the pathogenesis of metabolic disorders and inflammatory disease in the cardiovascular system. Chemerin regulates energy metabolism, adipogenesis, and angiogenesis [12,13,14] and plays a role in adaptive and innate immunity, acting as a chemoattractant for immune cells [15,16]. Systemic chemerin levels positively correlate with obesity-related phenotypes, such as body mass index (BMI), insulin resistance, and serum triglycerides, suggesting its function in metabolic diseases [16]. In addition, it has been suggested that chemerin levels determine the severity of coronary lesions since a positive correlation was observed between the presence of coronary artery disease and serum chemerin levels [17,18]. High chemerin levels are an independent predictor of coronary artery disease [19]. It was observed that plasma chemerin levels were increased in patients with coronary artery disease and were associated with an increased risk of significant adverse cardiovascular effects in these patients [19]. Chemerin secretion in the perivascular tissue correlates positively with aortic and coronary atherosclerosis [20], and chemerin has been linked to peripheral arterial stiffness [21], inflammation markers, and metabolic syndrome components [22] have been reported. This review focuses on the mechanisms of chemerin action and its role in the pathogenesis of CVDs. Furthermore, we discuss novel approaches for developing chemerin-targeting therapeutic agents to treat CVDs.

### Search Strategy

We searched PubMed and MEDLINE for English and non-English articles with English abstracts published between 1997 (when the first report on chemerin identification was published) and 2022. The top search terms were: chemerin, cardiovascular disease, chemerin receptors, adipokine, inflammation, endothelial dysfunction, and chemerin-targeting therapeutic agents. The search retrieved original peer-reviewed articles, which were further analyzed, focusing on the role of chemerin in CVDs. We specifically focused on including the most recent findings published in the past five years.

## 2. Cardiovascular Disease (CVDs)

The pathogenesis of CVDs is predominantly of atherosclerotic origin and progressively leads to the development of coronary artery disease, cerebrovascular disease, venous thromboembolism, and peripheral vascular disease, ultimately causing myocardial infarction, cardiac arrhythmias, or stroke [23]. Atherosclerosis is a progressive inflammatory disease characterized by lipid deposition in the arteries [24]. A defining event in the initiation of atherosclerosis is the subendothelial accumulation of low-density lipoprotein (LDL) which is exposed to the oxidative waste of vascular cells and modified to oxidized LDL (OxLDL) [25]. The recruitment of macrophages accompanies this process to fatty deposits on blood vessel walls [26] and ingestion of OxLDL by macrophages resulting in the formation of so-called “foam cells”, lipid-laden macrophages with a foamy appearance [27,28]. Foam cells secrete substances involved in plaque formation and its progression to more complex forms that can inhibit blood flow. Plaque growth can lead to the formation of a blood clot, resulting in myocardial infarction or stroke. Hypertension represents one of the most important risk factors for developing atherosclerotic heart disease, stroke, and peripheral artery disease. The pathogenesis of hypertension is characterized by progressively increased arterial stiffness, inflammation accompanied by activation of the renin–angiotensin–aldosterone system, and endothelial dysfunction [29].

Different behavioural and risk factors may increase inflammatory stress, leading to CVDs [30]. In this context, inflammation is a cause and aggravating factor in CVDs and a mediator of the disease’s worst prognosis. Recent studies have extensively examined the role of inflammation in the genesis and progression of CVDs [31,32]. However, new inflammatory biomarkers, such as C-reactive protein (CRP) [33], interleukins (IL) [34], tumour necrosis factor-alpha (TNF-α), and nitrotyrosine, have emerged in recent decades [30]. We must mention sirtuins (SIRT), micro RNAs (miRs), ST2 protein, apolipoprotein E protein, adiponectin, and others among these new biomarkers [30]. These biomarkers are preferentially expressed locally in the inflammatory target tissue, but they are also released into the peripheral blood and used as diagnostic and prognostic biomarkers [30]. Indeed, these biomarkers may predict future adverse cardiovascular events and a poor prognosis in CVDs patients. Furthermore, these new inflammatory biomarkers can be used to assess therapeutic efficacy in CVDs patients and could pave the way for new and exciting research into the relationship between inflammation and CVDs [30].

In obese patients, visceral fat and superficial adipose tissue are active endocrine tissues that express cytokines that can communicate with the cardiovascular system [35,36]. These cytokines, which include TNF, IL6, and anti-apoptotic proteins such as SIRT, are crucial in regulating adipose tissue function. Indeed, cytokine overexpression causes adipose tissue local dysfunction characterized by increased inflammation and oxidative stress, which is linked to a decrease in mitochondrial biogenesis [36]. In this context, SIRT is an NAD+-dependent deacetylase that regulates mitochondrial function, energy metabolism, adipocyte hypertrophy, cardiac regeneration, and cardiac remodelling [36,37]. Currently, altered glucose homeostasis induces the upregulation of inflammatory cytokines, which is associated with SIRT1 downregulation in obese diabetes patients. However, in diabetic patients versus normoglycemic patients, this may affect cardiovascular functions, resulting in altered myocardial performance and the development of heart failure [36]. According to Sardu et al., baseline hyperglycemia and insulin resistance are associated with higher expression of serum inflammatory cytokines and nitrotyrosine and lower expression of SIRT1 in subcutaneous abdominal fat in pre-diabetic patients versus normoglycemic patients [36].

Pre-diabetics have blood glucose and glycated haemoglobin levels that are not within the normal range for diabetes diagnosis [36]. Surprisingly, pre-diabetics have a higher risk of developing myocardial dysfunction, CVDs, and heart failure and an increased risk of death from any cause. As a result, altered glucose homeostasis appears to be the key factor influencing these molecular changes and clinical outcomes in pre-diabetic vs. normoglycemic patients [36]. In contrast, obesity-related changes in inflammation, oxidative stress, and cardiac cellular growth may impact myocardial function [36]. As a result, this pathological condition may be induced and exacerbated in obese patients with prediabetic conditions vs. patients with normoglycemic conditions due to altered glucose homeostasis. As a result of altered glucose homeostasis, this pathological condition may be induced and exacerbated in obese patients with prediabetic conditions versus patients with normoglycemic conditions [36]. Nitrotyrosine is produced by the oxidation of tyrosine and is an indicator of oxidative stress in overweight and diabetic patients [38]. Indeed, hyperglycemia is directly involved in the subsequent formation of nitrotyrosine in diabetic patients. Furthermore, patients with prediabetic conditions had a statistically significantly lower baseline value of SIRT1, downregulated by altered glucose homeostasis and linked to altered myocardial performance [36]. These inflammatory/oxidative molecular pathways were linked to various echographic changes. Patients with prediabetes had higher intima-media thickness values at baseline than obese patients with normoglycemic conditions [36]. Intima-media thickness is a significant atherosclerotic risk marker due to an adaptive response to changes in flow, wall tension, or lumen diameter [36,39]. On the other hand, higher intima-media thickness could be caused by non-atherosclerotic processes such as smooth muscle cell hyperplasia and fibro-cellular hypertrophy, which can result in medial hypertrophy and compensatory arterial remodelling [36]. Obese patients with prediabetes, on the other hand, may have anatomic and physiologic changes consistent with early arterial disease [36,37]. These molecular inflammatory/oxidative alterations were linked to higher values of septum thickness, posterior wall, left ventricle mass, and MPI at baseline in patients with pre-diabetic versus normoglycemic conditions. [36].

MicroRNAs (miRs) are important regulators of inflammation, adipose tissue function, and SIRT1 expression and are implicated in regulating insulin resistance and glucose homeostasis [40,41,42,43,44,45]. miRs are small endogenous non-coding RNAs that regulate gene expression by repressing translation or degrading target mRNAs [40,41,42,43,44,45]. miR-195 and miR-27, in particular, have been linked to adipose tissue and systemic inflammation, as well as SIRT1 expression, and are differentially expressed in overweight and normoglycemic vs. hyperglycemic patients. [42,43,44,45]. These effects resulted in significant reductions in intima-media thickness (IMT), left ventricular mass (LVM), and myocardial performance index (MPI) and an improvement in the left ventricular ejection fraction (LVEF). In obese pre-diabetic patients, miR-195 and miR-27 may regulate the expression of inflammatory/oxidative metabolites. Then, through the different regulations of miR195 and miR-27 expression, these molecules could influence the IMT, LVM, LVEF, and MPI. Indeed, at 12 months of follow-up, metformin therapy vs. placebo may significantly reduce inflammatory/oxidative stress and the expression of circulating miR-195 and miR-27 in pre-diabetic obese patients [45]. These effects resulted in significant reductions in IMT, LVM, and MPI and an improvement in LVEF. In this context, metformin therapy, in addition to a hypocaloric diet vs. placebo, appears to be an appropriate treatment to reduce hyperglycemia and insulin resistance and reverse systemic inflammation/oxidative stress in obese pre-diabetics via downregulation of circulating miR-195 [45]. Metformin therapy and a hypocaloric diet vs. placebo appear to be appropriate treatments to reduce hyperglycemia and insulin resistance and reverse systemic inflammation/oxidative stress in obese pre-diabetics via downregulation of circulating miR-195 and miR-27. Future research is required to evaluate the effects of metformin in pre-diabetic obese patients and its possible correlation with clinical outcomes via miRs modulation.

Metabolic diseases are closely linked to the development of CVDs and represent another significant global health problem associated with a high risk for morbidity and mortality [46]. Although metabolic diseases is an “umbrella term” for multiple related disorders, the prevalence of abdominal obesity, dyslipidemia, diabetes, and insulin resistance (IR) is observed [46,47]. The disbalance between energy intake and expenditure and genetic predisposition leads to the emergence of an obese phenotype [47,48]. Single mutations in genes coding for adipokines, such as leptin and its receptors, were shown to predispose the development of obesity [49,50]. An important factor associated with excessive fat accumulation is decreased blood supply, causing hypoxia and local fat tissue inflammation [51], gradually progressing into systemic inflammation [52]. Persistent activation of various inflammatory mediators leads to the development of IR, T2DM, and CVDs [53,54,55]. These diseases are accompanied by dyslipidemia [56] which is characterized by elevated total cholesterol, low-density lipoproteins (LDL), triglycerides (TG), very low-density lipoprotein (VLDL), a reduced level of high-density lipoprotein cholesterol (HDL-C), and decreased NO bioavailability [57,58,59,60,61,62]. Dyslipidemia induces endothelial dysfunction and initiates CVDs [57,58,59,63]. It has been shown that even moderately elevated cholesterol levels may be linked with CVDs [58,59]. Fat accumulation increases the release of inflammation-related factors such as macrophages and adipocyte-derived adipokines, further stimulating cytokine expression that perturbs metabolic homeostasis, promoting IR and hypertension [64,65,66,67]. Adipokines such as adiponectin, leptin, resistin, TNF-α, and various ILs affect vascular function by increasing the expression of angiotensin and endothelin and inhibiting NO production [68,69]. Disturbed lipid metabolism results in the accumulation of reactive oxygen species (ROS), leading to oxidative stress that augments cytokine production [70] and increases the amount of OxLDL, which is more physiologically harmful than LDL [69,71,72].

## 3. Chemerin

Chemerin is a small (18 kDa) protein that regulates numerous biological processes such as adipogenesis, glucose homeostasis, tumourigenesis, inflammation, angiogenesis, myogenesis, and immune cell migration [73,74]. The chemerin-encoding gene is known as retinoic acid receptor responder 2 gene (RARRES2) or tazarotene-induced gene 2 (TIG2) since it was initially discovered in psoriatic skin lesions exposed to the synthetic anti-psoriatic retinoid tazarotene [75]. Later it was found that human hepatocytes and adipocytes represent the major sites of chemerin synthesis [76,77], although significant expression of the RARRES2 gene was also observed in other tissues, such as kidneys, pancreas, adrenal glands, lungs, and skin [78,79,80]. Chemerin expression in various tissues may be either constitutive or regulated [13], and it is presumed that these two distinct pathways are differently controlled [81]. For example, adipocytes and hepatocytes have high constitutive RARRES2 mRNA levels [80], in contrast to immune cells such as monocytes and granulocytes in which the chemerin transcript is not detectable [82]. Observed differences in chemerin expression in different cell and tissue types may be of great importance for various pathological conditions such as obesity, cancer, inflammation, and cardiovascular diseases [83,84,85]. It has been proposed that chemerin expression may be regulated in a tissue-specific manner by metabolic and inflammatory mediators [86], including glucose, fatty acids, insulin, immunomodulatory cytokines, and agonists of nuclear receptors such as glucocorticoids, retinoids, and vitamin D [81]. Chemerin promoter analysis has revealed the presence of response elements for the peroxisome proliferator-activated receptor γ (PPARγ), sterol regulatory element-binding protein 2 (SREBP2), and farnesoid X receptor (FXR) [87,88,89], which are regulated by lipids (PPARγ), free fatty acids (SREBP2), and bile acids (FXR). Although the details of the molecular mechanism that directs the regulated expression of chemerin in different tissues are still not fully elucidated, epigenetic modifications such as DNA methylation were implied as a decisive factor affecting constitutive and regulated chemerin expression. DNA methylation in promoter regions is commonly associated with transcriptional repression, while methylated cytosines in CpG dinucleotides located within the gene are usually associated with transcriptional activation. A recent study demonstrates that DNA methylation has an important role in the cell-specific expression of RARRES2 in adipocytes, hepatocytes, and B lymphocytes [81]. The DNA methylation of RARRES2 controls the constitutive expression of chemerin, whereas acute-phase cytokines, interleukin 1b (IL-1β) and oncostatin M (OSM) were shown to regulate chemerin expression in a cell type-dependent manner [81].

**Figure 1:**
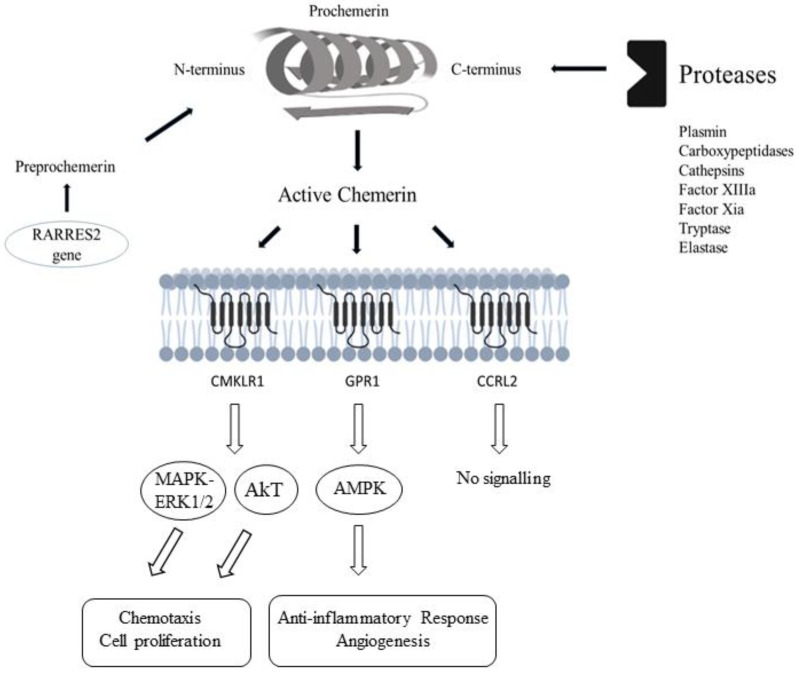
Chemerin synthesis and receptor signalling. RARRES2 retinoic acid receptor responder 2; CMKLR1 chemokine-like receptor 1; GPR1 G protein-coupled receptor 1; CCRL2 C-C chemokine receptor-like 2. Generated with Biorender.com.

Initially produced as preprochemerin, chemerin is then cleaved into prochemerin, an inactive precursor chemerin isoform that freely circulates in plasma. Active chemerin isoform is produced via post-translational processing, which entails the removal of 20 amino acids from the C-terminal of prochemerin by a variety of serine and cysteine proteases, such as plasmin, carboxypeptidases, cathepsins, Factor XIIIa, and Factor X (Figure 1) [74,77,78,90,91]. The original chemerin isoform consists of 163 amino acids, while other isoforms have different numbers relative to the original isoform’s length and include chemerin 125, 152, 154, 155, 156, 157, and 158 [78,92]. Generally, in humans, the chemerin circulating form is not bioactive, i.e., its bioactivity is determined by isoforms. The bioactivity of chemerin isoforms is defined by chemotaxis analyses (migration assays in CMKLR1 expressing cells) and intracellular calcium flux evaluation. According to the literature data, the Chem157 isoform has remarkable activity, followed by Chem158, Chem156, and Chem155 in the blood, synovium, skin, and adipocytes, while Chem125, Chem144, Chem152, Chem154, and Chem162 represent inactive chemerin isoforms [16,74,78,79,92]. In most studies on serum and plasma samples, the most abundant isolated form of chemerin was the precursor (Chem163), produced by the liver and secreted into the circulation [93,94,95]. The activation of Chem163 might appear through inflammation, coagulation, or fibrinolysis processes [77,96]. Considering the blood vessels, chemerin, as a full-length recombinant peptide, triggers artery contractions in both human and animal models [97,98,99]. Chemerin activation is moderated via three receptors: chemokine-like receptor 1 (CMKLR1, also known as Chem23), G protein-coupled receptor 1 (GPR1), and C-C chemokine receptor-like 2 (CCRL2) (Figure 1) and their mutual interactions are in the nanomolar range [76,100].

The interaction between chemerin and its receptors is important for various cellular and signalling mechanisms in the cardiovascular (CV), nervous, and reproductive systems [101,102,103]. CMKLR-1is the main chemerin receptor, and the chemerin/CMKLR1 axis promotes chemotaxis of natural killers (NK), macrophages, and dendritic cells [104]. GPR1 has a comparably similar affinity to chemerin as CMKLR1 with a similar sequence identity, and in experimental animal models, GPR1 participates in the development of adiposity, hormone secretion, and regulation of glucose equilibrium in obesity [102,105]. Interestingly, GPR1 plays a double role in chemerin activity, acting as both a signalling receptor via arrestin and a scavenger receptor for peptides that cannot stimulate receptor activation [106]. Although chemerin is the only recognized ligand for CCRL2, its interaction does not initiate ligand scavenging or chemotaxis [107,108]. The human population’s physiological level of plasma chemerin is approximately 50 ng/mL [109].

### 3.1. Chemerin and CVDs

An increasing body of evidence shows that chemerin plays numerous important roles in regulating the cardiovascular system and pathogenesis of CVDs, acting as an adipokine, chemoattractant, and growth factor. As an adipokine, chemerin modulates glucose and lipid levels, thus affecting lipid deposition in the endothelium [12,110] and the progression of atherosclerosis [12]. Chemoattraction is another prominent role of chemerin that enables the interaction of macrophages with dendritic cells and natural killer cells, directing them towards locations where damage occurs [79,111,112]. Chemerin promotes calcium mobilization and chemotaxis of immature dendritic cells and macrophages in the vasculature system [79], changes endothelial adhesion levels [113], and induces endothelial angiogenesis [114,115]. As a growth factor, chemerin promotes microcirculatory vessel outgrowth to sustain adipocyte aggregation and regulates osteoblastogenesis of bone marrow-derived precursor cells [100,115]. CMKLR1 receptor has been identified on the endothelium and smooth muscle layers of blood vessels, indicating chemerin’s considerable part in hypertension development since it moderates vascular tone and smooth muscle contractions [97,116]. Chemerin decreases NO-induced vascular relaxation and cyclic guanosine monophosphate (cGMP) formation [117,118]. In endothelial cells, NO is mainly produced by endothelial nitric oxide synthase (eNOS) [119]. Chemerin was found to decrease eNOS generation and stimulate NO breakdown, resulting in overall NO reduction in endothelial cells. It has been speculated that additional mechanisms, such as eNOS uncoupling and reduced NO-dependent cGMP signalling, could contribute to chemerin-mediated endothelial dysfunction [117,120].

Chemerin’s role in the proliferation and migration of endothelial cells central to developing atherosclerosis is well documented. Chemerin promotes angiogenesis by stimulating the endothelial cells’ proliferation while simultaneously functioning as their chemoattractant [121] in a process that depends on p38 mitogen-activated protein kinase (MAPK) and the extracellular regulated protein kinases (ERK) 1/2 pathway in human umbilical vein endothelial cells (HUVEC) [114,121,122]. Chemerin also exerts a dose-dependent effect on matrix metalloproteinases-2/9-mediated extracellular matrix degradation, further affecting the endothelial cells’ proliferation and migration capacity [123].

Chemerin was shown to increase the expression of various endothelial inflammatory factors such as IL-6, TNFα, and CRP, which results in abnormal endothelial secretion, blood vessel wall inflammation, and increased monocyte attachment to endothelial cells [124,125,126]. Interestingly, inflammatory cytokines such as TNF-α, IL-1β, and IL-6 were found to modulate chemerin effects by increasing the expression of CMKLR1 receptor in endothelial cells [120]. In human coronary artery endothelial cells, elevated circulating chemerin concentration is associated with increased expression of intercellular adhesion molecule 1 (ICAM-1) and E-selectin, which are regarded as typical markers of vascular endothelial activation [127]. Based on the literature data, it could be suggested that inflammatory mechanisms mediate many effects of chemerin that lead to endothelial dysfunction. Chemerin is also associated with excessive ROS accumulation in endothelial cells, contributing to endothelial dysfunction [124]. Mitochondrial ROS production was increased upon treatment of human aorta endothelial cells with chemerin, whereas treatment with a ROS scavenger N-acetylcysteine or knockdown of CMKRL1 receptor led to inhibition of ROS production [128]. These data suggest that chemerin exerts effects on mitochondria, which represent the major source of intracellular ROS production and are further supported by findings that the mitochondria-targeted antioxidant Mito-TEMPO suppressed the chemerin-mediated ROS generation [128].

Chemerin’s effects on vascular smooth muscle cells (VSMCs) are extensively studied. The proliferation and migration of VSMCs are involved in vascular remodelling, and the abnormal vascular structure is accompanied by vascular dysfunction [129]. Studies show that short-term in vitro treatment of VSMCs with chemerin (20 min) increased the proliferation and migration capacity of VSMCs via MAPK and Akt/ERK signalling [130,131], the endothelin-1 dependent pathway [132], and increased autophagy [133]. Interestingly, prolonged incubation of VSMCs with chemerin (6 h) led to VSMC’s apoptosis [126], suggesting that chemerin may exert different functions at different stages of vascular remodelling and dysfunction [120]. In addition, increased ROS accumulation and elevated expressions of inflammatory cytokines such as IL-1β, IL-6, and monocyte chemoattractant protein-1 (MCP-1) were observed in chemerin-treated VSMCs, suggesting that chemerin can induce VSMC dysfunction by augmenting oxidative stress and promoting inflammation [126,134,135,136,137].

The current state of knowledge points to the role of chemerin, a global regulatory protein that mediates a variety of cardiovascular functions. In the following section, we present further important evidence establishing a connection between chemerin and CVD pathogenesis as evidenced by human and animal studies.

#### 3.1.1. Evidence from Human Studies Supporting Chemerin’s Role in CVDs

Chemerin’s significant role in vascular dysfunction processes is illustrated by in vitro findings that chemerin stimulation increases reactive oxygen species (ROS) generation and inflammation in human microvascular endothelial cells and VSMCs [126]. Chemerin participates in endothelial inflammation via pro-inflammatory transcriptional regulator NF-_K_B activation and increases monocyte–endothelial adhesion [125]. According to recent research, chemerin serum levels are positively associated with unstable plaques and blood vessel disorders. [138,139]. A broad spectrum of chemerin’s associations with atherosclerosis is possibly due to its interference with macrophage activity via its CMKLR1 receptor [140,141]. Serum chemerin levels, for example, correlated with atrial remodelling and fibrillation, blood pressure, lipid status, and BMI in a cross-sectional study of male and female human patients with atrial fibrillation [142]. In a prospective cohort study with 834 patients, chemerin was labelled as a novel serum biomarker for predicting major adverse cardiac disorders in chronic heart failure [143]. According to data from another cohort study, plasma chemerin levels increased in male and female patients with progressive carotid stenosis and correlated positively with different inflammatory markers, indicating chemerin’s influence on atherosclerosis [144]. Various in vivo studies validated the correlation between increased chemerin levels and obesity and metabolic syndrome [85,145]. Still, we should be cautious about plasma chemerin concentrations since it involves assays that distinguish between chemerin and prochemerin isoforms. In addition to chemerin’s positive association with visceral adiposity and insulin resistance (IR), chemerin also correlates with carotid intima-media thickness, suggesting its potential role in CV risk evaluation [146] (Table 1).

In patients with rheumatoid arthritis, chemerin levels are independently associated with atherosclerosis plaque formation and endothelial function [141]. In addition to adults and elderly patients, CV risk prevalence could be perceived in younger populations [150,151], and some authors indicated that chemerin levels in obese and diabetic adolescents positively correlate with various biochemical parameters related to CV risk occurrence [147,148]. However, in another study on human subjects where serum chemerin levels were positively associated with various cardiometabolic risk factors, such as triglyceride, fasting glucose, coronary artery stenosis, and others, multiple regression indicated that chemerin does not represent an independent factor of risk for multiple vessel disorders [149].

Interestingly, some authors state that chemerin participates in pre-eclampsia development by CMKLR1/Akt/enhancer-binding protein-alpha (CEBPα) axis activation and angiogenesis suppression and induces M1 macrophage polarization [152]. Expression levels of chemerin, pregnancy-associated plasma protein A (PAPP-A), ox-LDL, and matrix metalloproteinase 9 were found to be independent risk factors for neurological impairment in ischemic cerebrovascular disease patients [153]. The chemerin/CMKLR1 axis promotes vascular smooth muscle cell migration and proliferation through Akt/ERK phosphorylation, causing vascular remodelling and hypertension [131] (Table 1). It was observed that adult patients with primary hypertension had significantly higher serum chemerin concentrations compared to healthy controls [154]. In addition, the concentration of circulating chemerin was increased in obese children with elevated systolic blood pressure [155,156]. Although numerous pieces of evidence show a strong positive correlation between chemerin concentration and blood pressure, further clinical studies are required to support the predictive potential of the association of chemerin concentration with hypertension.

Recently, it has been proposed that the local tissue chemerin concentrations, not the circulating chemerin levels, are responsible for controlling blood pressure [78]. In particular, adipocytes in the fat tissue were suggested to be a source of biologically relevant chemerin for blood pressure regulation. According to this suggestion, perivascular adipose tissue (PVAT) facilitates the local action of chemerin in the vasculature by serving as a source of chemerin that may activate CMKLR1 receptors in sympathetic nerves and/or smooth muscle cells to stimulate vascular contraction [78,157].

This view is supported by findings that chemerin may enhance sympathetic nerve function in rats where PVAT-produced chemerin amplified superior mesenteric arterial contraction induced by electrical-field stimulation (EFS) via activation of CMKLR1 receptor [98]. The experiments suggest sympathetic nerve stimulation may lead to chemerin secretion [78]. Furthermore, exogenous chemerin-9 potentiated EFS-induced arterial contraction, which is important in light of the sympathetic nervous system’s role in controlling and regulating blood pressure. Also, high chemerin mRNA transcripts are found in the adrenal gland [82], which is part of the sympathetic nervous system and exerts blood pressure control via epinephrine and mineralocorticoids. Chemerin release in the adrenal medulla provoked by sympathetic nerve activation would activate the receptors in the adrenal cortex, representing another possible mechanism for blood pressure control by increased local concentrations of chemerin.

Chemerin was also associated with an abdominal aortic aneurysm (AAA), representing progressive abdominal aortic dilation. The concentration of circulating chemerin was increased in patients with AAA, and the analysis of abdominal aortic samples from AAA patients revealed increased mRNA levels of both chemerin and CMKRL1 relative to healthy controls, suggesting the involvement of chemerin/CMRKL1 axis in AAA pathogenesis and progression [158]. The protein expression of chemerin and CMKLR1 receptor was also analyzed by immunohistochemistry in human aortas, coronary vessels, and periadventitial adipose tissue (PVAT) and strongly correlated with the presence of atherosclerosis. Chemerin immunopositivity was observed in PVAT, VSMCs, and foam cells in atherosclerotic lesions, whereas CMKLR1 was expressed in VSMCs and foam cells in aortic and coronary vessels with atherosclerotic lesions. Although chemerin and CMKLR1 protein expression significantly correlated with the severity of aortic atherosclerosis [140], chemerin is not recommended as an atherosclerosis marker due to the dependence of its predictive potential on the location of affected arteries and the disease stage [22,159,160]. However, chemerin may be considered a predictor of acute coronary syndrome (ACS) since the concentration of circulating chemerin was significantly higher in patients with ACS relative to those with stable angina pectoris and healthy controls, and the increase in chemerin concentration strongly correlated with the elevation of CRP concentration [154,161,162].

#### 3.1.2. Chemerin Roles in CVDs: Evidence from Animal Studies

In diabetes, the incidence of cardiomyopathy, ischemia, and micro/macrovascular dysfunction is increased [163]. Chemerin partially participates in glucose homeostasis, inducing IR in rat cardiomyocytes via the ERK1/2 pathway (Table 2) [164]. Diabetic retinopathy is a frequent and early microvascular complication [165]. According to Jun et al., chemerin through CMKLR1 induces ICAM-1 expression and vascular endothelial growth factor (VEGF) secretion in rats’ primary retinal microvascular endothelial cells, stimulating the angiogenic process in diabetes pathology [166]. In a study on cultured cardiomyocytes of rats, data showed that chemerin suppresses Akt phosphorylation and caspase-9 activation and consequently leads to cell apoptosis [167]. In cardiac fibroblasts isolated from Wistar rats, chemerin-9, an active fragment of chemerin, induced cell migration, and ROS increase [168]. Previous studies showed that chemerin affects adipocyte differentiation and moderates different long non-coding RNAs (IncRNAs) and micro RNAs (miRNAs) responsible for fat accumulation and VEGF expression and activity, such as lncRNA Meg3 and miR-217 [169]. A recent study on female chemerin knockout rats reported data regarding chemerin’s role in adipocyte growth in mesenteric fat [170]. In addition, chemerin modified blood pressure in chemerin knockout (KO) female rats, but this effect was not observed in male rats, suggesting a possible sex dependency [171]. In the same animal model, it is shown that chemerin affects sympathetic nerve-mediated contraction and vascular tone [172]. Chemerin’s association with the progression of atherosclerosis was investigated by manipulating chemerin levels in vivo. Adenovirus transfection was used to knockdown or overexpress the chemerin gene in the aorta or plasmacytoid dendritic cells (pDCs) of apolipoprotein (Apo) E^−/−^ mice on a high-fat diet [173,174]. This approach revealed that chemerin accelerates the progression of atherosclerosis [174]. In addition, the knockout of CMKLR1 receptors in pDCs of ApoE^−/−^ mice restricted the formation and progression of atherosclerotic plaque [173]. It has been suggested that the pro-atherosclerotic effect of chemerin can be explained by increased adhesion and migration of endothelial cells [110], the proliferation of VSMCs, and inflammation [175]. The human and animal studies presented in this review strongly support the conclusion that chemerin dysregulation represents a risk factor for CVDs and obesity.

## 4. Perspectives for the Development of Chemerin-Targeting Therapeutic Agents

The increasing amount of data on chemerin’s role in the pathogenesis of CVDs gave rise to an intriguing possibility of using chemerin and associated signalling proteins, such as receptor CMKLR1, as targets for developing novel therapeutic agents for the management of CVDs. One of the most extensively studied candidates is the compound CCX832, a CMKLR1 inhibitor shown to significantly ameliorate chemerin-induced vascular dysfunction in vitro and in vivo [120].

CCX832 has been shown to reduce chemerin-induced vascular inflammation in human microvascular endothelial cells, ameliorate consequences of oxidative stress in human aortic smooth muscle cells, and reverse chemerin-induced angiogenesis via decreased expression of P38 MAPK, ERK1/2, and matrix metalloproteinases-2/9 [99,120,176]. In addition, CCX832 exerted an inhibitory effect on the abnormal contraction of human pulmonary and coronary arteries [98,177]. The observed effects of CCX832-mediated inhibition of CMKLR1 were further confirmed in experimental studies using RNA interference by short hairpin RNA (shRNA) to suppress CMRKL1 expression [178]. Data showed that CMRKL1 knockdowns were associated with reversed angiogenesis, reduced oxidative stress and downregulated expression of autophagy-related genes [121,128,179]. Despite promising experimental results and phase 1 clinical trial initiation in patients with psoriasis, the development of CCX832 was discontinued in February 2012 for unknown reasons. However, research on CCX832 has paved the way for identifying and developing other CMKLR1 inhibitors that should have similar effectiveness in ameliorating chemerin-induced vascular dysfunction and improving the safety profile in humans.

Another ligand for binding to CMKLR1 is resolvin E1 (RvE1), a member of the family of compounds derived from omega-3 polyunsaturated fatty acids (PUFA). RvE1 is a specialized pro-resolving mediator (SPM) with a potent immunomodulatory role in the resolution of inflammation. In addition, RvE1 has a crucial role in reducing chemerin-mediated vascular dysfunction and associated CVDs risk [180,181], most probably through competing with chemerin for the binding site of CMKLR1. RvE1 was shown to have numerous benefits for the cardiovascular system, such as regulation of vasoconstriction [98], inhibition of atherosclerotic plaque progression [182], and restriction of vascular calcification [183].

Several synthetic fragments of chemerin were shown to possess biological activity, acting as chemerin analogues and agonists of CMKLR1. For instance, chemerin-9 (C9) has an anti-inflammatory role, reducing TNF-α levels and decreasing the areas of atherosclerotic aortic lesions [184]. C9 was recently shown to attenuate abdominal aortic aneurism formation in ApoE^−/−^ mice by significantly suppressing the infiltration of inflammatory cells, neovascularization, and matrix metalloproteinase expression, while increasing the presence of elastic fibres and smooth muscle cells (SMCs) [185]. However, C9 may also adversely affect the cardiovascular system by inducing arterial contraction and hypertension [184]. Further studies addressing the efficacy and safety of C9 and other synthetic chemerin fragments are required to assess their potential for therapeutic use. Regarding other synthetic fragments of chemerin, some authors pointed out that Chem156 also reflects an anti-inflammatory role in synovial fluid of patients with arthritis and experimental hepatocellular carcinoma [186,187].

Finally, antisense oligonucleotides (ASOs) targeting the chemerin gene by sequence-specific binding were also investigated in animal studies, and it was demonstrated that chemerin knockout by ASO results in a significant decrease in blood pressure [188]. These findings open up a perspective on the possible use of chemerin-targeting ASOs for the treatment of hypertension.

## 5. Conclusions

Evidence points to chemerin’s crucial role in CVD’s development and progression. As an adipokine, chemerin modulates glucose and lipid levels, thus affecting lipid deposition in the endothelium and the progression of atherosclerosis. As a chemoattractant, it facilitates the mobilization and interaction of macrophages with dendritic cells and natural killer cells in the vasculature system and induces endothelial angiogenesis. The established chemerin’s role in vascular inflammation, angiogenesis, and blood pressure modulation opens up exciting perspectives for developing chemerin-targeting therapeutic agents for the treatment of CVDs. Several candidates that target chemerin and the CMRK1 signalling pathway have shown promising potential in reducing vascular dysfunction in numerous in vitro and in vivo studies. Further research addressing the efficacy and safety of novel chemerin-targeting agents is required to assess their potential therapeutic application.

## Figures and Tables

**Table 1:** Chemerin and CVDs: Evidence from human studies.

**Patient’s Gender and Age (Mean ± SD or Median and Range)**	**CVDs**	**Chemerin Levels**	**CV- Associated Disorders/Parameters and Chemerin Correlation**	**Reference**
male and female 11.6 ± 2.0	↑ BMI	↑ (serum)	BMI, waist circumference, leptin, body fat insulin, HDL-C and TC	[85]
male and female 48.4 ± 10.9	dyslipidemia, hypertension	↑ (plasma)	*RARRES2* gene polymorphism, hs-CRP	[138]
male and female 43.5 ± 13.0	rheumatoid factor-positive, hypertension	↑ (plasma)	Hs-CRP, leptin, vascular adhesion molecule, monocyte chemoattractant protein	[141]
male and female 60.54 ± 9.64	arterial fibrillation	↑ (serum)	arterial fibrillation, BMI, SBP, DBP, TC, LDL-C, creatinine, hs-CRP and left atrial diameter	[142]
male and female 66 (58–75)	hypertension, chronic heart failure, diabetes, hyperlipidemia	↑ (serum)	heart failure, diabetes, hs-CRP, hypertension	[143]
male and female 66.9 ± 0.6	coronary artery disease	↑ (plasma)	TC, hsCRP, peripheral leukocyte count, TNF-α	[144]
male and female 45.5 (18–69)	↑ BMI, impaired glucose tolerance	↑*RARRES2* expression (whole blood)	visceral fat mass	[145]
male and female 44.0 ± 10.1	hypertension, diabetes, ↑ BMI	↑ (plasma)	waist circumference, HOMA-IR, fat mass, HbA1c, cIMT	[146]
male and female 16.3 ± 1.5	atherosclerotic lesions and cardiac autonomic neuropathy, diabetes type 1	↑ (serum)	vaspin and LDL-C	[147]
female 13.9 ± 1.8	↑ BMI	↑ (serum)	TG, HDL-C, LDL-C and fat mass	[148]
male and female 62.2 ± 10.0	coronary stenosis, hypertension, diabetes	↑ (serum)	fasting glucose, TC, LDL-C, hs-CRP, degree of coronary artery stenosis	[149]
**Cell Culture Model**	**Chemerin Concentrations**	**Duration of Stimulation**	**CV-Associated Disorders/Parameters–Chemerin Correlation**	**Reference**
human microvascular endothelial cells	10 nM	2 h	↑ endothelial cell adhesion, protein expression and secretion, activates NF-_K_B	[125]
human microvascular endothelial and vascular smooth muscle cells	50 ng/mL	5, 15, 30, 60 min and 2, 8, 24 h	↑ O_2_·^−^, ↑ H_2_O_2_, ↑ *Nox* 1, ↑ *Nox* 4 and ↑ miRNA expression, ↑ phosphorylation of SAPK/JNK and ERK1/2, ↓ eNOS, ↓ NO and apoptosis	[126]
human peripheral blood mononuclear cells	2.5, 25, 50 and 100 ng/mL	12, 24, 36 and 48 h.	↑ adhesion and migration abilities of endothelial progenitor cells	[138]

↑ increased; ↓ decreased; BMI, body mass index; cIMT, carotid intima-media thickness; CV, cardiovascular; CVDs, cardiovascular disorders; DBP, diastolic blood pressure; eNOS, endothelial nitric-oxide synthase; ERK, extracellular signal-regulated kinase; H_2_O_2_, hydrogen peroxide; HbA1c. glycated haemoglobin; HDL-C. High-density lipoprotein cholesterol; HOMA-IR, Homeostatic Model Assessment for Insulin Resistance; hsCRP, high sensitive c-reactive protein; LDL-C, low-density lipoprotein cholesterol; NO, nitric oxide; Nox, NADPH oxidases; O_2_·^−^, superoxide anion; SAPK/JNK, stress-activated protein kinases/jun amino-terminal kinases; SBP, systolic blood pressure; TC, total cholesterol; TG, triglycerides; TNF-α tumour necrosis factor α.

**Table 2:** Chemerin and CVDs: Evidence from animal studies.

**Animals (Gender)**	**Tissues**		**CV-Associated Disorders/Parameters–Chemerin Correlation**	**Reference**
chemerin knockout Sprague Dawley rat (female)	thoracic aorta		blood pressure modification	[171]
chemerin knockout Sprague Dawley rat (female)	plasma, mesenteric adipocytes		↓ visceral adiposity	[170]
chemerin knockout Sprague Dawley rat (female)	superior mesenteric arteries		↓ vascular tone	[172]
**Cell Culture Model**	**Chemerin Concentrations**	**Duration of Stimulation**	**CV-Associated Disorders/Parameters–Chemerin Correlation**	**Reference**
rat vascular smooth muscle cells	1–300 ng/mL	24 h	↑ vascular smooth muscle cells proliferation and migration	[131]
Sprague Dawley rat’s cardiomyocytes	10 and 100 ng/mL	24 h	impaired insulin signalling and induced insulin resistance	[164]
Sprague Dawley rat’s cardiomyocytes	0.1, 1, 10 and 100 nM	6–48 h	cardiomyocytes apoptosis	[167]
mouse 3T3-L1 preadipocytes	0, 20, 40, 60, 80 and 100 ng/mL	48 h	miRNA-217 suppression (correlated with fat accumulation), induced preadipocytes differentiation into adipocytes, ↑ Meg3 lncRNA	[169]
Wistar rat’s cardiac fibroblasts	100 ng/mL	12 h	fibroblast migration, ↑ ROS	[168]

miRNA, micro RNA; Meg3, maternally expressed gene; lncRNA, long non-coding RNA; ROS, reactive oxygen species.
